# Sodium–Glucose Cotransporter Inhibitors: Cellular Mechanisms Involved in the Lipid Metabolism and the Treatment of Chronic Kidney Disease Associated with Metabolic Syndrome

**DOI:** 10.3390/antiox13070768

**Published:** 2024-06-26

**Authors:** Fernando Cortés-Camacho, Oscar René Zambrano-Vásquez, Elena Aréchaga-Ocampo, Jorge Ismael Castañeda-Sánchez, José Guillermo Gonzaga-Sánchez, José Luis Sánchez-Gloria, Laura Gabriela Sánchez-Lozada, Horacio Osorio-Alonso

**Affiliations:** 1 Doctorado en Ciencias Biologicas y de la Salud, Universidad Autónoma Metropolitana, Mexico City 04960, Mexico; fcortesc1700@alumno.ipn.mx (F.C.-C.); renezambrano2513@gmail.com (O.R.Z.-V.); 2 Departamento de Fisiopatología Cardio-Renal, Instituto Nacional de Cardiología Ignacio Chávez, México City 14080, Mexico; jose.gonzaga@cardiologia.org.mx (J.G.G.-S.); laura.sanchez@cardiologia.org.mx (L.G.S.-L.); 3 Departamento de Ciencias Naturales, Universidad Autónoma Metropolitana, Unidad Cuajimalpa, Mexico City 05348, Mexico; earechaga@cua.uam.mx; 4 Departamento de Sistemas Biológicos, Universidad Autónoma Metropolitana, Unidad Xochimilco, México City 04960, Mexico; jcastanedas@correo.xoc.uam.mx; 5 Department of Internal Medicine, Division of Nephrology, Rush University Medical Center, Chicago, IL 60612, USA; jose_sanchez@rush.edu

**Keywords:** metabolic syndrome, obesity, hypertension, insulin resistance, dyslipidemia, lipid metabolism, lipotoxicity, oxidative stress, chronic kidney disease, sodium–glucose cotransporter 2 inhibitors

## Abstract

Metabolic syndrome (MetS) is a multifactorial condition that significantly increases the risk of cardiovascular disease and chronic kidney disease (CKD). Recent studies have emphasized the role of lipid dysregulation in activating cellular mechanisms that contribute to CKD progression in the context of MetS. Sodium–glucose cotransporter 2 inhibitors (SGLT2i) have demonstrated efficacy in improving various components of MetS, including obesity, dyslipidemia, and insulin resistance. While SGLT2i have shown cardioprotective benefits, the underlying cellular mechanisms in MetS and CKD remain poorly studied. Therefore, this review aims to elucidate the cellular mechanisms by which SGLT2i modulate lipid metabolism and their impact on insulin resistance, mitochondrial dysfunction, oxidative stress, and CKD progression. We also explore the potential benefits of combining SGLT2i with other antidiabetic drugs. By examining the beneficial effects, molecular targets, and cytoprotective mechanisms of both natural and synthetic SGLT2i, this review provides a comprehensive understanding of their therapeutic potential in managing MetS-induced CKD. The information presented here highlights the significance of SGLT2i in addressing the complex interplay between metabolic dysregulation, lipid metabolism dysfunction, and renal impairment, offering clinicians and researchers a valuable resource for developing improved treatment strategies and personalized approaches for patients with MetS and CKD.

## 1. Introduction

Metabolic syndrome (MetS) constitutes a cluster of interconnected disorders, encompassing central obesity, dyslipidemia, hypertension, a prothrombotic and proinflammatory state, insulin resistance (IR), and hyperglycemia. This intricate interplay of metabolic derangements significantly heightens the susceptibility to developing type 2 diabetes mellitus (T2DM) and cardiovascular disease (CVD) across both macrovascular and microvascular beds, including chronic kidney disease (CKD) [1,2,3].

MetS has proliferated into a global health crisis, afflicting children and adults worldwide. Its prevalence varies markedly based on diverse factors such as geographical location, age, gender, ethnicity, lifestyle choices, and diagnostic criteria employed. Clinically, the diagnosis of MetS hinges on the simultaneous presence of multiple disorders, commonly referred to as risk factors (RFs). The widely recognized diagnostic criteria, such as those delineated by the National Cholesterol Education Program Adult Treatment Panel III (NCEP ATP III), the World Health Organization (WHO), and the International Diabetes Federation (IDF), typically incorporate a constellation of three to five RF (Table 1) [2,4].

The global prevalence of metabolic syndrome (MetS) is not uniform, as it is influenced by both diagnostic criteria and geographical location. Recent research indicates a wide range in worldwide prevalence from 12.5% to 31.4%, highlighting the impact of different definitions and assessment methods. Notably, the United States has observed a higher prevalence, with 35.6% of women and 30.3% of men affected between 2003 and 2012. This variation underscores the importance of considering both methodological and regional factors when interpreting MetS prevalence data [5,6]. In this regard, it was observed that, among patients aged over 60 years, more than 50% were women of Hispanic ancestry [6]. According to the results of the Encuesta Nacional de Salud y Nutrición (ENSANUT), in Mexico from 2006 to 2018, the increase in the prevalence of MetS was 20%; in men, the increase in the prevalence of MetS was 18.09, and in women, it was 22.23%. In that study, the most prevalent RF was abdominal obesity at 74, 72, 78, and 81% in 2006, 2012, 2016, and 2018, respectively [7]. MetS has emerged as a significant contributor to mortality, and its impact increases with the number of RFs present and the severity of MetS components. Notably, as the complexity of MetS rises, so does the patient’s susceptibility to various forms of mortality, including all-cause mortality, heart disease-related mortality, and mortality linked to diabetes mellitus (DM) [8]. Conversely, among both women and men aged 30 to 39 years, there has been a notable 25% increase in the prevalence of hypertension with advancing age, which directly affects the 30% increase in mortality rates attributable to this condition [9].

Multiple studies have indicated that the presence of three or more risk factors significantly elevates the likelihood of developing CKD, with an increased risk of up to 130%. Notably, even in the early stages of CKD, disruptions in lipid metabolism can emerge, manifesting as renal dysfunction and microalbuminuria, a key indicator of kidney damage. This underscores the importance of early detection and intervention in managing lipid metabolism disorders to mitigate the progression of CKD [10,11].

On the other hand, while sodium–glucose cotransporter 2 inhibitors (SGLT2i) have demonstrated promising effects on various MetS components, their potential in mitigating CKD associated with MetS remains under-explored. Therefore, this comprehensive review aims to bridge this gap by elucidating the intricate relationship between RFs, lipid metabolism impairment, and subsequent renal dysfunction. We also delve into the therapeutic potential of SGLT2i in addressing these interconnected processes, examining the systemic effects, molecular targets, and nephroprotective mechanisms of SGLT2i. This review provides a detailed overview of how individual, or combined MetS risk factors contribute to lipid metabolism dysfunction, leading to lipid accumulation and renal damage. Moreover, we highlight the pleiotropic effects of SGLT2i, including their impact on body weight, dyslipidemia, IR, and hyperglycemia—all key components of MetS. This review also discusses the approved SGLT2i, their mechanisms of action, and their observed nephroprotective effects. Additionally, we discuss the global impacts, other well-known effects including cardioprotective benefits, and the effects of SGLT2i when used in co-therapy with other antidiabetic drugs. The findings underscore the potential of SGLT2i as a therapeutic strategy for CKD associated with MetS. By targeting multiple RFs and their downstream effects on lipid metabolism, SGLT2i offers a promising avenue for mitigating renal dysfunction and improving patient outcomes. This review serves as a valuable resource for clinicians and researchers, encouraging further investigation into the therapeutic applications of SGLT2i beyond diabetes management.

## 2. Methodology

This manuscript critically reviews the current knowledge on CKD associated with MetS and metabolic dysfunction. Additionally, we review the use of SGLT2 inhibitors as therapy for metabolic diseases. This manuscript consolidates the findings from multiple studies to provide a comprehensive understanding of the intricate relationship between metabolic syndrome, lipid metabolism disorders, and CKD. We conducted an electronic search through PubMed, Scopus, and Google Scholar to find the most suitable studies. The search used keywords and a combination of sentences that included them. The search terms utilized, without limitation, were “SGLT2i, insulin resistance, metabolic dysfunction, mitochondrial function, dyslipidemia, oxidative stress, and lipotoxicity”. The articles included in this review were considered according to the following criteria: 1.—clinical and experimental studies about kidney disease, metabolic syndrome, hypertension, obesity, dyslipidemia, insulin resistance, hyperglycemia, mitochondrial dysfunction, lipotoxicity, and metabolic dysfunction; 2.—interventional studies conducted on human, cell, and animal models (diabetes, obesity, hypertension, metabolic syndrome) using SGLT2 inhibitors focusing on CKD; 3.—articles published in the English language.

The articles selected involved an initial screening: The titles and abstracts of all retrieved articles were analyzed for relevance based on the inclusion criteria. Subsequently, the full texts of potentially relevant articles were obtained and assessed for eligibility. Finally, data were extracted from the eligible studies, including the type of pathology and experimental model used, interventions administered, outcomes assessed, and key findings.

## 3. Risk Factors Involved in Target Organ Damage

MetS pathogenesis results from a complex interplay of genetic, dietary, sedentary lifestyle, environmental, cultural, psychological, and societal factors. The interrelation of RFs with organ damage is triggered by cellular mechanisms that activate signaling pathways, including vasoactive, inflammatory, fibrotic, and pro-oxidant pathways, leading to damage to cells, tissues, organs, and systems, including the kidneys [2]. Furthermore, systemic or localized alterations can exacerbate and perpetuate the damage, creating a feedback loop that intensifies the severity of the condition.

Therefore, from a therapeutic standpoint, gaining a deeper understanding of the cellular and molecular mechanisms driving lipid accumulation, renal dysfunction, and CKD development in MetS is crucial.

In this context, we aim to address the pivotal RFs implicated in ectopic lipid accumulation and impaired lipid metabolism, along with their roles in renal injury during MetS. We will discuss obesity, dyslipidemia, insulin resistance, mitochondrial dysfunction, and reactive oxygen species (ROS) as key contributors in the renal lipotoxicity induced by MetS. Additionally, we will focus on the emerging therapeutic potential of SGLT2i in controlling these RFs.

### Obesity, Dyslipidemia, and Insulin Resistance as Pathological Mechanisms

Some factors such as an unhealthy diet, a sedentary lifestyle, genetic predisposition, and medical conditions like hypothalamic, iatrogenic, or endocrine disorders contribute to body weight gain, also known as obesity, which is classified according to established criteria [12]. The classification of weight-related conditions often relies on body mass index (BMI), with overweight defined as a BMI of 25 to less than 30 kg/m^2^ and obesity defined as a BMI of 30 kg/m^2^ or higher, according to the WHO guidelines [13]. In Mexico, according to ENSANUT 2018, 74.2% of Mexican adults are overweight (39%) or obese (36%), and 82% have abdominal adiposity [14]. Furthermore, obesity increases the risk of T2DM, nonalcoholic fatty liver disease (NAFLD), and hypertension [15,16].

In both overweight and obese individuals, excess body weight manifests as the accumulation of adipose tissue (AT), which can exist in two primary forms, white adipose tissue (WAT) or brown adipose tissue (BAT), each serving distinct functions [15]. WAT is predominantly found in subcutaneous deposits around the abdomen and visceral areas, producing adipokines and factors that regulate energy balance and obesity, including leptin; adiponectin; angiotensinogen; and cytokines such as interleukin-1beta (IL-1β), interleukin-6 (IL-6), interleukin-8 (IL-8), interleukin-10 (IL-10), tumor necrosis factor-alpha (TNF-α), chemokines, and profibrotic substances like transforming growth factor-beta (TGF-β) [17,18]. These molecules play crucial roles in metabolic regulation, inflammation, and tissue remodeling, contributing to the complex interplay observed in obesity-related pathology [15], i.e., leptin is crucial in promoting the esterification of fatty acids (FA) into triglycerides (TG) while enhancing lipolysis. Conversely, adiponectin exerts insulin-sensitizing effects by boosting fatty acid oxidation (FAO) and simultaneously lowering TG and FFA serum levels [19].

**Figure 1:**
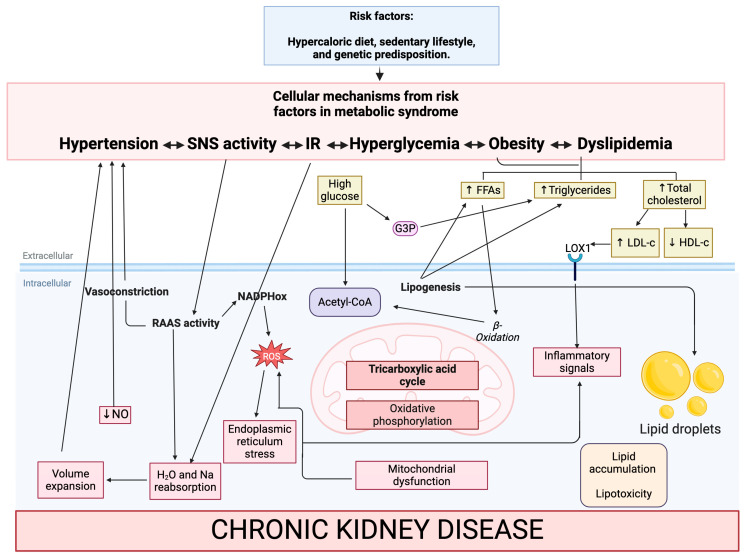
Cellular mechanisms from risk factors in metabolic syndrome and their role in kidney damage. Metabolic dysfunction alters the lipid profile, leading to the activation of inflammatory processes, oxidative stress, and increased lipid accumulation in the circulation and renal tissue. Insulin resistance and dyslipidemia deteriorate mitochondrial function, favoring ROS production and cell apoptosis. On the other hand, SNS hyperactivity promotes renal sodium reabsorption, leading to hypertension and renal injury. Additionally, the high activity of RAAS contributes to ROS and affects renal hemodynamics, sodium retention, and vasoconstriction, which causes kidney damage. Abbreviations: FFAs, free fatty acids; G3P, glyceraldehyde 3-phosphate; HDL-c, high-density lipoprotein cholesterol; LDL-c, low-density lipoprotein cholesterol; SNS, sympathetic nervous system; ROS, reactive oxygen species; RAAS, renin–angiotensin–aldosterone system; LOX1, lectin-like oxidized low-density lipoprotein receptor-1; NO, nitric oxide. IR, insulin resistance; NADPHox, nicotinamide adenine dinucleotide phosphate oxidase.

Irrespective of the specific adipose tissue type, AT functions as a dynamic organ dispersed throughout the body, managing surplus energy intake and storage through enlarging existing adipocytes (hypertrophy) or generating new ones (hyperplasia). However, when its capacity is surpassed, there is a swift escalation in the circulation of free fatty acids (FFA) (Figure 1) [18,20]. Additionally, metabolic imbalance and the limited capacity of AT to increase proportionally in response to energy storage may lead to ectopic fat distribution in other organs, such as the heart, liver, skeletal muscle, pancreas, and kidney [18].

On the other side, obesity is frequently associated with an impairment in the lipid profile (dyslipidemia), including alterations in TG, total cholesterol (TC), high-density lipoprotein cholesterol (HDL-c), and low-density lipoprotein cholesterol (LDL-c) [21].

The prevalence of dyslipidemia and hypertriglyceridemia (HTG) has risen in the population over recent years. A study reported that the prevalence rates of HTG were 29.6% in the global population, 36.9% in men, and 23.8% in women, while low levels of HDL-c were observed in 78% to 81% of cases [22,23]. In this context, HDL-c is essential for promoting cholesterol efflux from cells through reverse cholesterol transport (RCT), reducing the size of atherosclerotic plaques, and playing an important role in lipid homeostasis [24]. Moreover, HDL-c plays a crucial role in cardiovascular protection by activating endothelial nitric oxide synthase (eNOS) and producing nitric oxide (NO), a key regulator of vascular function. HDL-c also exhibits antioxidant and anti-inflammatory properties, contributing to its cardioprotective effects by removing and inactivating oxidized LDL (ox-LDL) and stimulating NO synthesis [24,25]. According to Kon et al., eNOS stimulation occurs through the binding of HDL-c to the scavenger receptor class B type I (SR-BI), which activates a tyrosine kinase (Src), subsequently activating phosphoinositide 3-Kinase (PI3K), serine/threonine kinase (Akt), and ras-mitogen-activated protein kinase (MAPK) pathways [26].

On the other hand, LDL-c particles consist of triglycerides and cholesterol esters encapsulated within an outer layer composed of phospholipids, free cholesterol, and apolipoprotein B (ApoB). This structural arrangement facilitates the transportation of hydrophobic cholesterol throughout the bloodstream. Elevated levels of LDL-c are strongly associated with the development of atherosclerosis [27]. Oxidized LDL-c (ox-LDL) is the primary driver of lipid accumulation and inflammation in the vascular wall, triggering and contributing to the progression of atherosclerosis. Even a mild increase in ox-LDL elevates the atherogenic risk [28,29]. In addition, elevated levels of LDL-c in CKD enhance the differentiation of monocytes to proinflammatory M1 macrophages via action on the lectin-like oxidized low-density lipoprotein receptor-1 (LOX-1) (Figure 1) [24].

Insulin stimulates glucose uptake in insulin-dependent tissues and inhibits lipolysis, thereby activating adipogenesis and lipogenesis through the promotion of the expression of sterol regulatory element-binding protein 1c (SREBP-1c) and related transcription factors involved in adipocyte differentiation [30]. In AT, insulin suppresses the breakdown of triglycerides into FFA by inhibiting hormone-sensitive lipase (HSL) [31]. Also, insulin regulates serum very-low-density lipoprotein cholesterol (VLDL-c) concentration by suppressing its synthesis and stimulating clearance [31].

The diminished response to insulin impairs glucose uptake, thus resulting in plasma glucose accumulation and hyperglycemia, hyperinsulinemia, and IR. This, in turn, promotes a prothrombotic state and amplifies the release of proinflammatory cytokines from AT [32,33]. IR increases the expression of lipogenic genes, such as acetyl-CoA carboxylase 1 (ACC1), fatty acid synthase (FAS), and glycerol-3-phosphate acyltransferase 1 (GPAT1), resulting in increased lipid synthesis and accumulation [34]. In an IR state, circulating FFA derived from adipocytes are elevated, inhibiting glucose absorption, glycogen synthesis, and glucose oxidation while increasing glucose production by the liver. Moreover, there is a correlation between increased FFA and decreased insulin-stimulated insulin receptor substrate (IRS-1) phosphorylation and PI3K activity. Thus, the accumulation of TG and its metabolites (diacylglycerol, fatty acyl-CoA, and ceramides) is closely linked to IR and circulating FFA [30]. In patients with both type 1 and type 2 DM, the lipolysis results in an elevated supply of FFA to the liver [35]. Consequently, IR contributes to the suppression of β-oxidation in patients with diabetic nephropathy (DN) and advanced CKD. This underscores the kidneys as crucial target organs for the deleterious effects of lipotoxicity mediated by obesity, insulin resistance, and adipose tissue dysfunction [36].

On the other hand, obesity impairs lipid metabolism and insulinemia through the activation of the sympathetic nervous system (SNS) and renin–angiotensin–aldosterone system (RAAS). This increases the generation of reactive oxygen species (ROS), renal sodium retention, impairment in diastolic relaxation, and a decrement in the levels of NO, contributing to endothelial dysfunction, hypertension, and kidney damage (Figure 1) [37,38,39].

## 4. Role of Lipids in Renal Damage

Impaired lipid homeostasis in MetS produces excess adipokines and FFA, resulting in the accumulation in non-adipose tissues such as the kidney, including podocytes, mesangial cells, tubular epithelial cells, and interstitial cells (Figure 1) [40,41]. Moreover, such effects increase oxidative stress, lipid peroxidation, mitochondrial dysfunction, inflammation, fibrosis, apoptosis [31], and glomerular and tubular dysfunction [42]. Histologically, CKD is characterized by glomerulomegaly, focal segmental glomerulosclerosis (FSGS), mesangial proliferation, and hypertrophied podocytes [40,43,44].

In patients with CKD, the lipid profile is characterized by HTG and changes in the structure and function of the lipoproteins [21,24]. According to Kon et al., the kidneys participate in the metabolism of HDL-c and its components through several mechanisms, including 1.—glomerular filtration rate, 2.—tubular uptake, 3.—catabolism, 4.—transport by the lymphatic vascular network in the interstitium, and 5.—urinary excretion [45,46]. Therefore, kidney dysfunction is associated with proteome changes in HDL-c. Thus, patients with CKD have reduced levels of apolipoprotein A1 (ApoA1), lecithin coenzyme cholesterol acyl-transferase (LCAT), and paraoxonase-1 (PON1), reducing the antioxidant function of HDL-c, while deficiency of LCAT is involved in the progression of CKD in individuals at an early stage of renal dysfunction [46]. In addition, the kidney contributes to HDL-c catabolism, being the main site of decay of ApoA1 and small HDL-c particles [46]. ApoA1 is essential for the efflux of cholesterol from peripheral cells and functions as LCAT [47]. According to Miljkovic M et al., patients undergoing dialysis exhibited biochemical findings revealing reduced levels of ApoA1 and HDL-c [48].

**Figure 2:**
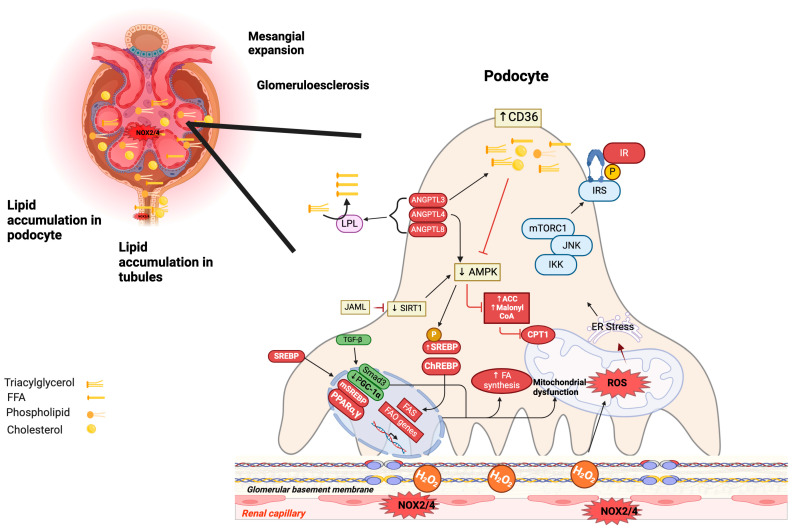
Role of lipids in chronic kidney disease. CD36 is responsible for transporting FFA in podocytes, and these are stored in lipid drops, which inhibits AMPK activity, increasing the levels of ACC and malonyl-CoA and inactivating the CPT1 enzyme important for FAS; under these conditions, lipid synthesis and lipotoxicity increase. SREBP1 and ChREBP proteins can induce overexpression of lipogenic genes that favor de novo lipogenesis. Overexpression of JAML in podocytes affects lipid accumulation via SIRT1-AMPK-SREBP1, causing renal dysfunction. ANGPTL3, ANGPTL4, and ANGPTL8 are key regulators of LPL, which can reduce triglyceride levels to FFA, resulting in hypertriglyceridemia and causing damage to the structure of podocytes and proteinuria. Alternately, TGF-β decreases the expression of PGC-1α and, through Smad3, activates the synthesis of fatty acids. Low expression of PPARα exhibits higher lipid accumulation, while elevated levels of PPARγ increase lipogenesis. On the other hand, excess cholesterol increases the production of ROS by NOX4/NOX2, increasing H2O2. OS from high concentrations of fatty acids in podocytes induces IR by activating PKC; NF-κB; and JNK. In addition, several kinases, such as IKKβ; ERK, and mTORC1, are activated, resulting in phosphorylation of IRS-1 at inhibitory sites leading to IR. The black arrow indicates stimulation and the red lines indicate inhibition. Abbreviations: ANGPTL3, angiopoietin-like 3; ANGPTL4, angiopoietin-like 4; ANGPTL8, angiopoietin-like 8; CD36, cluster of differentiation 36; FFA, free fatty acids; AMPK, AMP-activated protein kinase; ACC, acetyl coenzyme A carboxylase; CPT1, carnitine palmitoyl transferase 1; SREBP-1, sterol regulatory element-binding protein 1; ChREBP, carbohydrate response element-binding protein; JAML, junctional adhesion molecule-like protein; SIRT1, sirtuin 1; LPL, lipoprotein lipase; TGF-β, transforming growth factor-beta; PGC-1α, peroxisome proliferator-activated receptor γ coactivator 1α; PPARα, peroxisome proliferator-activated receptor alpha; PPARγ, peroxisome proliferator-activated receptor gamma; ROS, reactive oxygen species; NOX4, NADPH oxidase 4; NOX2, NADPH oxidase 2; H_2_O_2_, hydrogen peroxide; IR, insulin resistance; PKC, protein kinase C; NF-κB, nuclear factor kappa B; JNK, c-Jun N-terminal kinase; IKKβ, nuclear factor kappa B kinase subunit beta; mTORC1, mammalian target of rapamycin.

Excess cholesterol increases the production of ROS by the nicotinamide adenine dinucleotide phosphate oxidase (NADPHox) or (NOX) pathway in glomerular, tubular, and tubulointerstitial cells. NADPHox 4 (NOX4) is highly expressed in kidney tubular epithelial cells and contributes to the production of hydrogen peroxide (H_2_O_2_) (Figure 2) [49].

Elevated levels of FA within podocytes trigger IR by phosphorylation of insulin receptor substrate 1 (IRS-1) protein (Figure 2). This increases ROS production and the activation of protein kinase C (PKC), nuclear factor-kappa B (NF-κB), inhibitor of NF-κB kinase subunit beta (IKKβ), c-Jun N-terminal kinase (JNK), extracellular signal-regulated kinase (ERK), and mammalian target of rapamycin (mTOR), leading to lipid accumulation, hypertrophy, glomerulosclerosis, and apoptosis, resulting in a decline in GFR (Figure 2) [34,40,48,50].

### 4.1. Cellular Mechanisms Involved in Kidney Lipotoxicity

The accumulation of lipids in kidney tissue involves the participation of signaling pathways responsible for uptake, synthesis, and transport. Evidence suggests that podocytes express proteins involved in cholesterol efflux, such as LDL receptor (LDLR), ATP-binding cassette transporter A1 (ABCA1), and 3-hydroxy-3-methyl-glutaryl CoA reductase (HMGCR), which contribute to lipid buildup within the kidneys, resulting in renal damage [51].

The cluster of differentiation 36 (CD36) is a multifunctional transmembrane receptor that mediates the binding and cellular uptake of long-chain FA, ox-LDL, and phospholipids into podocytes, mesangial and microvascular endothelial cells, macrophages, and proximal and distal tubular epithelial cells. Thus, its overexpression is associated with an increased FFA uptake; particularly, an increased palmitic acid uptake leads to a dose-dependent increase in the levels of mitochondrial ROS, depolarization, adenosine triphosphate (ATP) depletion, and activation of apoptosis (Figure 2) [52,53,54,55].

Recent studies have highlighted a group of proteins crucial for regulating cell polarity, epithelial barrier formation, and leukocyte migration and their involvement in lipid metabolism [56,57]. Among these proteins is junctional adhesion molecule-like protein (JAML), representing a novel JAM family member. JAML regulates podocyte lipid metabolism through the Sirtuin 1 (SIRT1)-mediated/SREBP1 signaling pathway (Figure 2) [57]. In patients and experimental models of CKD with FSGS, JAML overexpression induces lipid accumulation. The lipogenic effects of JAML in damaged podocytes were likely mediated through the SIRT1–AMPK–SREBP1 pathway (Figure 2) [57]. Likewise, JAML deficiency resulted in greater SIRT expression and ras-mitogen-activated protein kinase (MAPK) activation and attenuation of lipid accumulation and renal dysfunction [57].

FAO requires fatty acids as substrates delivered by LPL activity, which is regulated by many extracellular proteins, including members of the angiopoietin-like family of proteins (ANGPTL) [58]. The angiopoietin-like 3 (ANGPTL3) [59], angiopoietin-like 4 (ANGPTL4) [60], and angiopoietin-like 8 (ANGPTL8) modulate LPL through inhibition of the function and dimerization of LPL, leading to a decrease in the conversion of TG to free FFA producing HTG (Figure 2) [59,61]. In ANGPTL3-knockout mice fed an HFD, there was a notable reduction in hyperlipidemia and proteinuria. This reduction was closely associated with a decline in podocin expression, indicating the potential role of ANGPTL3 in hyperlipidemia-associated podocyte injury [62]. ANGPTL3 activates integrin β3 and rac family small GTPase 1 (RAC1) in podocytes, triggering the generation of ROS, cytoskeletal rearrangement, and podocyte motility through autocrine or paracrine signaling pathways, ultimately proteinuria [62]. In mice with an HFD, the absence of ANGPTL4 showed a significant reduction in hyperlipidemia and proteinuria. Additionally, the structure of podocytes is preserved under these conditions [63]. Qiu et al. reported that treatment with palmitic acid increases the expression of ANGPTL4 in podocytes and causes injury through the AMPK/ACC signaling pathway [64].

SREBP-1 is a transcription factor that regulates the expression of both anabolic and catabolic proteins in lipid metabolism. Its overexpression leads to aberrant lipid accumulation in renal tubular cells [65,66]. In experimental models of kidney damage, SREBP-1 overexpression increases FA and TG deposition through FAS overexpression, which leads to glomerulosclerosis, apoptosis, loss of tubular brush border, and proteinuria [66,67,68].

Other hallmarks of MetS include hyperglycemia and an excess of energy. Carbohydrate response element-binding protein (ChREBP) plays a significant role in lipid accumulation in diabetic kidneys. In both experimental diabetes models and cell cultures of renal tubular epithelial cells, high glucose levels lead to increased expression of acetyl-CoA synthetase 2 (ACSS2), oxidative stress, inflammation, and fatty acid synthesis. The underlying cell mechanism involves the ACSS2/SIRT1/ChREBP signaling pathway [69,70]. In advanced stages of CKD, there is an increase in ChREBP, FAS, and ACC, downregulation of the peroxisome proliferator-activated receptor-alpha (PPARα)-regulated fatty acid oxidation system, and reduction of diacylglycerol acyltransferases (DGAT), resulting in HTG [71]. Meanwhile, ChREBP deficiency inhibited fatty acid synthesis and mTORC1 activity and, in turn, upregulated the expression of PPARα, CPT1A, and acyl-coenzyme A oxidase 1 (ACOX1), thereby reducing triglycerides and renal lipid accumulation and improving renal function [72].

Another mechanism contributing to lipid accumulation and renal diseases involves autophagy, which is responsible for clearing dysfunctional organelles and recycling cellular components [73]. Autophagy is regulated by nutrient and oxygen deprivation and involves hypoxia-inducible factor-1-alpha and 2-alpha (HIF-1α and HIF-2α), AMPK, and SIRT1. In the kidney, AMPK plays a key role in lipid metabolism and autophagy, suppressing oxidative stress, inflammation, and fibrosis [74]. AMPK regulates fatty acid oxidation and cholesterol synthesis through phosphorylation and deactivation of 3-hydroxy-3-methylglutaryl-CoA reductase (HMGCR), an essential enzyme involved in cholesterol synthesis [74].

In obese mice subjected to endurance exercise training, AMPK-mediated phosphorylation of ACC and UNC51-like kinase 1 (ULK-1) was enhanced, leading to increased FAO and autophagy but decreased lipid accumulation, tubulointerstitial fibrosis (TGF-β, Col I, and Col III), oxidative stress, and inflammation [TNFα, IL-1β, IL-6, and monocyte chemoattractant protein-1 (MCP-1)] in the kidney [75]. In HFD-fed mice, the inhibition of AMPK increases fibrosis and inflammation in the kidney, possibly mediated by the regulation of NOX, TGF-β, and NF-kB [76,77].

A group of nuclear factors involved in metabolism belongs to the peroxisome proliferator-activated receptors (PPARs), which are represented by three subtypes: peroxisome proliferator-activated receptor alpha (PPARα), peroxisome proliferator-activated receptor delta (PPARδ), and peroxisome proliferator-activated receptor gamma (PPARγ) [78]. PPARα is expressed in glomerular and mesangial cells, podocytes, the proximal tubules, as well as the thick ascending limb [79]. In experimental models lacking PPARα, elevated levels of blood glucose, free fatty acids, and triglycerides in plasma are observed along, with lipid accumulation in the kidneys. Interestingly, this contrasts with lower FAO in the kidney compared to wild-type models, and these mice exhibit albuminuria, inflammation, and fibrosis [80,81]. PPARγ is expressed in the glomeruli, including the juxtaglomerular apparatus, podocytes, and medullary interstitial cells [81]. Animal models deficient for PPARγ show alterations in renal lipid metabolism and develop severe kidney damage demonstrated by inflammation, interstitial fibrosis, glucosuria, and albuminuria [80,81].

### 4.2. The Role of Mitochondria in Lipid-Induced Renal Damage in Metabolic Syndrome

In the healthy kidney, proximal tubule cells rely heavily on lipid oxidation for ATP production, utilizing β-oxidation in the mitochondria. However, under conditions like hyperglycemia, impaired glucose tolerance, or dyslipidemia, renal metabolic profiles change, increasing intracellular oxidation, but the lipid metabolism in the mitochondria is disrupted. This shift stimulates renal gluconeogenesis for ATP synthesis, resulting in tubular atrophy, interstitial fibrosis, ectopic lipid accumulation, and lipotoxicity [82].

Fatty acids entering cells are converted into fatty acyl-CoA by acyl-CoA synthase (ACS) and then transported into the mitochondria via carnitine palmitoyl-transferase 1 (CPT-1) [31]. Carnitine acyl-carnitine translocase (CACT) facilitates acyl-carnitine translocation across the inner mitochondrial membrane, while carnitine palmitoyl-transferase 2 (CPT-2) inside the mitochondria regenerates acyl-CoAs by removing carnitine from acyl-carnitines. Acetyl-CoA, a key intermediate, is generated through β-oxidation and is a vital substrate in metabolic pathways [83,84]. Acetyl coenzyme A carboxylase (ACC) regulates FAO by converting acetyl CoA to malonyl-CoA, which inhibits CPT-1. Low ACC activity increases fatty acid entry into the mitochondria, enhancing FAO. This tightly regulated process ensures efficient energy production and metabolic balance in renal tubule cells [85].

In experimental models of CKD and dyslipidemia, HTG and increased lipogenesis were observed and related to a decrease in renal mitochondrial β-oxidation [69,86,87,88]. The renal and mitochondrial dysfunctions were associated with increased FFA uptake, the expression of CD36, fatty acid-binding protein 4 (FABP4), SREBP, FAS, ACC, and stearoyl-CoA desaturase-1 (SCD1), which are involved in FFA biosynthesis. Conversely, those proteins related to FFA oxidation, such as PPARα, CPT1, mitochondrial ATPase α chain (ATP5a), acyl-CoA oxidase 1 (ACOX1), peroxisome proliferator-activated receptor γ coactivator 1α (PGC-1α), voltage-dependent anion-selective channel proteins 1 VDAC1, SIRT1, and phosphorylated AMP-activated protein kinase (pAMPK), were decreased. These changes were associated with an increase in ROS production and lipid droplet accumulation in renal cells, while the markers of mitochondrial biogenesis were decreased [69,86,87,88].

The heterotrimeric complex AMPK plays a crucial role in maintaining mitochondrial homeostasis and optimizing oxidative phosphorylation. It enhances mitochondrial biogenesis by phosphorylating at least two key regulators: PGC1α, via activation of SIRT1, and transcription factor EB (TFEB) [86]. AMPK regulates enzyme activity by phosphorylating ACC, which inhibits its function. This leads to decreased intracellular levels of malonyl-CoA and increased FAO. As a result, there is a reduction in lipid synthesis and a decrease in lipotoxicity within the cell (Figure 2) [74,86]. In contrast, excess acetyl-CoA increases the production of malonyl-CoA through the activity of ACC, which inhibits CPT1 function and leads to a reduction in the mitochondrial influx of fatty acyl-CoA. This activation of FAS and lipogenesis results in the synthesis of palmitate stored as TG [31]. PGC-1α has been identified as the primary upstream transcriptional regulator of mitochondrial biogenesis and function in the kidney [89].

## 5. Treatment of Chronic Kidney Disease Induced by Lipotoxicity

Throughout this manuscript, we have reviewed the pivotal role of renal lipid metabolism as a driving force behind renal dysfunction and damage. Evidence suggests that factors such as hyperglycemia, IR, obesity, and dyslipidemia favor lipid synthesis and deposition, impair lipid oxidation, and activate oxidative, profibrotic, and proinflammatory pathways, contributing to the development of glomerulosclerosis and fibrosis in the kidney. Therefore, interventions aimed at controlling, modulating, or improving these RFs and even restoring lipid metabolism through dietary counseling, lifestyle changes, and medications hold promise for kidney protection. This approach could be especially beneficial in contexts where obesity, dyslipidemia, and IR are prevalent.

In this regard, traditional medicine has served as an initial therapeutic approach and, whether used independently or as an adjuvant, could prove valuable in managing RFs associated with MetS [90,91]. While the effectiveness of herbal medicine remains a topic of ongoing debate due to factors such as variations in preparation, quality control, and limited clinical trials, its significant contributions to modern medicine should not be overlooked. Many isolated compounds derived from plants, fruits, vegetables, and spices, which possess demonstrated biological activity and beneficial effects, have served as models or precursors for developing innovative drugs currently used in clinical practice [92,93]. This is exemplified by sodium–glucose cotransporter inhibitors (SGLTi), a class of antidiabetic drugs renowned for their efficacy in managing risk factors associated with MetS. These inhibitors trace their origins to phlorizin, a natural compound isolated from the apple tree. We briefly review the studies with herbal medicine that addressed the inhibition of SGLTs aimed at beneficial effects, thus offering a therapeutic option in the treatment of MetS RFs.

### 5.1. Traditional Medicine Utilizing Sodium–Glucose Cotransporter Modulation as an Alternative Therapy for RFs

Using a COS-7 cell line model, researchers found that the ethanolic extract of *Schisandrae chinensis fructus* (SCF) derived from the fruit of *Schisandra chinensis* effectively inhibited the activity of both sodium–glucose cotransporter 1 (SGLT1) and sodium–glucose cotransporter 2 (SGLT2), as measured by a [^14^C]-α-methyl-D-glucopyranoside ([^14^C]-AMG) uptake assay. The inhibition rates were notable, with 89% for SGLT1 and 73% for SGLT2. However, the specific compounds responsible for this inhibitory effect within the SCF extract remain unidentified [94]. Furthermore, a study on hamsters fed a high-fat diet (HFD) revealed that SCF ethanolic extract effectively lowers serum levels of total cholesterol, TG, and LDL-c, alleviating IR, inflammation, and lipid accumulation in the liver. That study demonstrated the potent hypolipidemic and anti-inflammatory properties of SCF and identified key lipid metabolic regulators involved in its mechanism of action, namely p-AMPK, CPT1, SREBP1c, and ACC [95]. Sato et al. reported that the methanolic extract of *Sophora flavescens* (Fabaceae) exhibits potent inhibitory activity on the function of SGLT1 and SGLT2 expressed in cell culture of COS-1 cells [96]. Other studies conducted on models genetically predisposed to type 2 diabetes, such as KK-Ay mice and Sprague–Dawley rats fed an HFD, have revealed the promising effects of *Sophora flavescens* treatment on glucose metabolism. Specifically, administration of *S. flavescens* resulted in a notable improvement in glucose tolerance, reduced hyperglycemia, and decreased insulin levels through modulation of the IRS/PI3K/AKT and IKK/NF-κB/TNFα pathways [97,98].

*Gnetum gnemonoides*, a tropical plant rich in stilbenoids, has yielded several compounds with biological activity, including resveratrol, oxyresveratrol, piceatannol, and isorhapontigen [99]. Shimokawa et al. assessed the inhibitory effects of *Gnetum gnemonoides* on SGLT2 function and identified two stilbene trimers, gneyulins A and B [100]. They also reported that the oxyresveratrol trimers, gneyulins A and B, exhibited mild inhibition on SGLT1 and SGLT2 activities [99,100].

**Figure 3:**
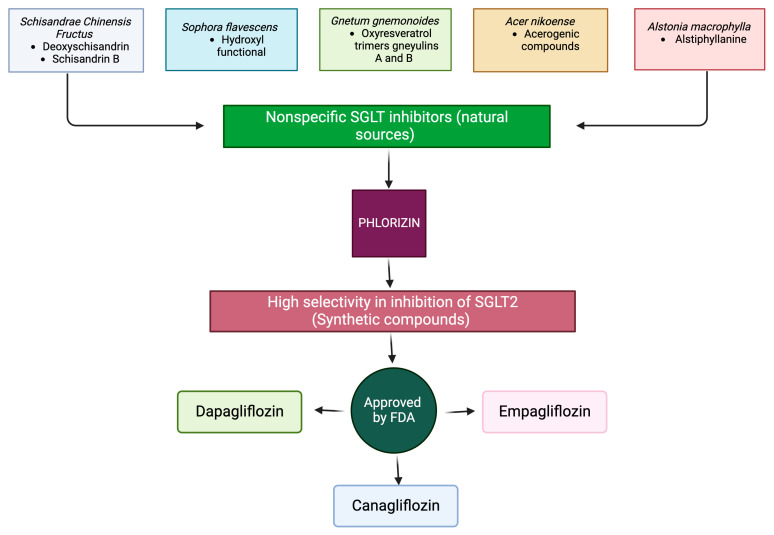
Natural source with reported non-specific SGLT inhibitor. *Schisandrae Chinensis Fructus*, *Sophora flavescens*, *Gnetum gnemonoides*, *Acer nikoense*, *Alstonia macrophylla*. Phlorizin, a natural compound, inhibits both SGLT1 and SGLT2. Several FDA-approved SGLT2 inhibitors, such as dapagliflozin, canagliflozin, and empagliflozin, have been synthesized with improved selectivity and specificity compared to phlorizin. Abbreviations: FDA, Federal Drug Administration; SGLT, sodium–glucose cotransporter; SGLT2, sodium–glucose cotransporter 2.

Morita et al. conducted a study using a methanol extract of *Acer nikoense* bark and identified two acerogenic compounds, A (1) and B (2), which exhibited significant inhibitory activity on SGLTs. Specifically, acerogenins A and B demonstrated potent inhibition of SGLT1, with percentages of 92.7% and 94.2%, respectively. Although their inhibitory effect on SGLT2 function was comparatively lower, at 33.9% and 54.2%, respectively, it still suggests a potential role in modulating glucose uptake (Figure 3) [101].

A methanolic extract of *Alstonia macrophylla leaves*, which is rich in the three alkaloids picralin, alstifilanin, and ajmaline, was used to assess the uptake of methyl-α-D-glucopyranoside (a glucose analog) in cultured cells expressing either SGLT1 or SGLT2. At a concentration of 50 µM, these alkaloids demonstrated moderate inhibitory activity against SGLT1 and SGLT2 [102].

While several plant extracts have shown inhibitory activity against SGLTs, their effectiveness has not yet reached the level of success achieved by the glycoside phlorizin. This naturally occurring compound, derived from the apple tree, has been extensively studied and utilized for its ability to control hyperglycemia, inflammation, oxidative stress, insulin resistance, and obesity. Phlorizin, a natural phenolic glycoside (glucopyranoside), serves as the basis for developing more effective and specific SGLT2 inhibitors. It is the glucoside of phloretin, a member of the dihydrochalcones family of bicyclic flavonoids (Figure 3). Phlorizin was first isolated from the apple tree in 1835 but can also be found in varying concentrations in other plants [103,104]. Phlorizin functions by blocking glucose absorption and reabsorption through selective and competitive inhibition of SGLT1 in the mucosa of the small intestine and in the late S2 and S3 segment of the proximal tubule in the kidney. Additionally, phlorizin inhibits SGLT2 activity in the early S1/S2 segment in the proximal tubule, reducing hyperglycemia and inducing high diuresis, glycosuria, and natriuresis [105,106]. In experimental models of diabetes, the administration of phlorizin has proven effective in the management of hyperglycemia, oxidative stress, and lipid disorders [103,105,107]. However, phlorizin does have certain limitations: (1) Lack of Selectivity: Phlorizin inhibits both SGLT1 and SGLT2 without therapeutic selectivity. While SGLT2 inhibition is desired for its glucose-lowering effects, inhibition of SGLT1 can lead to undesirable gastrointestinal side-effects due to its role in intestinal glucose absorption. (2) Adverse gastrointestinal effects: Phlorizin’s inhibition of SGLT1 in the intestines can cause diarrhea, dehydration, and malabsorption. These side-effects significantly affect patient compliance and limit the therapeutic use of phlorizin. (3) Low oral bioavailability: Phlorizin is poorly absorbed in the small intestine, resulting in low oral bioavailability; therefore, higher doses are needed to achieve therapeutic concentrations, potentially exacerbating gastrointestinal side-effects [99,103].

Despite showcasing numerous beneficial properties in the context of metabolic diseases, phlorizin has not progressed to clinical use. However, phlorizin’s value lies in serving as a precursor for modifying and synthesizing multiple compounds. Some of these derivative compounds have already received approval from the Food and Drug Administration (FDA), while others are currently undergoing clinical trials (Figure 3) [99].

### 5.2. Sodium–Glucose Cotransporter 2 Inhibitors (SGLT2i) as an Emergent Treatment for MetS

The kidney plays a crucial role in glucose homeostasis with its intricate filtration, reabsorption, and secretion functions. Glucose, filtered by the glomeruli, is predominantly reabsorbed in the proximal tubule via SGLTs. Thus, SGLT2 is responsible for reabsorbing up to 90% of filtered glucose to maintain systemic balance [108,109]. This significant reabsorption process highlights the significance of targeting SGLT2 as a therapeutic approach for managing glycemia in diabetes [109]. SGLT2i, such as ipragliflozin, canagliflozin, and others, have emerged as promising agents in this regard, effectively reducing hyperglycemia by inducing glycosuria through blocking the reabsorption of glucose and sodium in the kidney [110,111].

**Figure 4:**
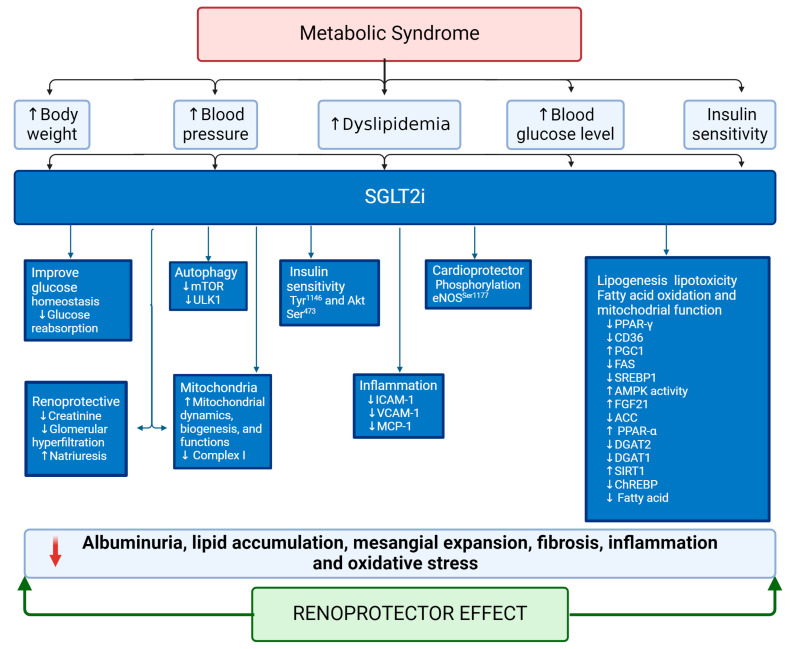
Role of SGLT2i in controlling risk factors in metabolic syndrome and the progression of renal damage. Abbreviations: SGLT2i, sodium–glucose cotransporter 2 inhibitor; iCAM-1, intercellular adhesion molecule-1; VCAM-1, vascular cell adhesion molecule-1; MCP-1, monocyte chemoattractant protein-1; mTOR, mammalian target of rapamycin; ULK1, unc-51 like autophagy activating kinase 1; eNOS, nitric oxide synthase; AKT, serine/threonine kinase; AMPK, AMP-activated protein kinase; Ser, serine; Tyr, tyrosine; ACC, acetyl coenzyme A carboxylase; SREBP1, sterol regulatory element-binding protein 1; PPARα, peroxisome proliferator-activated receptor alpha; PPARγ, peroxisome proliferator-activated receptor gamma; CD36, cluster of differentiation 36; PGC1, proliferator-activated receptor γ coactivator 1α; FAS, fatty acid synthase; FGF21, fibroblast growth factor 21; DGAT2, diacylglycerol O-acyltransferase 2; DGAT1, diacylglycerol O-acyltransferase 1; SIRT1, sirtuin 1; ChREBP, carbohydrate response element-binding protein.

Beyond their primary role in glycemic control, SGLT2i exhibit a spectrum of beneficial effects. They improve insulin resistance and facilitate weight loss. At the renal level, they mitigate hyperfiltration and albuminuria, thereby delaying the progression of renal disease [108,110,112]. Additionally, SGLT2i induce a natriuretic effect, leading to osmotic diuresis. Combined with glycosuria, this effect reduces plasma volume and blood pressure, offering cardioprotective effects (Figure 4) [108,111,112,113]. Furthermore, SGLT2i induces uricosuria, providing additional cardiovascular and renal protection [114,115,116]. In patients with MetS, including obesity, type 2 diabetes mellitus (T2DM), and related conditions, SGLT2i has significantly reduced various metabolic parameters, such as body weight, blood pressure, and lipid profile. These improvements contribute to preserved renal function and natriuresis, highlighting the broader metabolic benefits of SGLT2i beyond glycemic control [108,112,113,117].

Moreover, studies have clarified the impact of SGLT2i on lipid metabolism and signaling pathways. For instance, treatment with dapagliflozin raises HDL-c levels and protects against atherosclerosis and atherothrombosis in both clinical studies and experimental models [118].

In obese mice on a high-fat diet, empagliflozin administration boosts insulin sensitivity by stimulating phosphorylation of the insulin receptor β subunit (Tyr1146) and Akt (Ser473) in WAT, liver, and muscle. This leads to increased AMP-activated protein kinase (AMPK) and ACC phosphorylation in skeletal muscle and higher hepatic and plasma levels of fibroblast growth factor 21 (FGF21), enhancing energy expenditure and heat production [119]. Elevated levels of DGAT2 in the liver facilitate the synthesis and storage of TG in lipid droplets. Conversely, canagliflozin reduces mRNA expression of DGAT2, PPAR-γ receptors, and SGLT2 while decreasing PPARα levels in the liver (Figure 4) [120]. Canagliflozin induces transcriptional reprogramming to activate catabolic pathways, increase fatty acid oxidation, reduce hepatic steatosis and diacylglycerol content, and elevate liver and plasma levels of FGF21, promoting lipolysis, ketogenesis, energy expenditure, and weight loss (Figure 4) [121]. Canagliflozin also suppresses liver lipid synthesis and the expression of ATP-citrate lyase, ACC, and SREBP-1c independently of AMPK-β1, affecting adiposity and energy expenditure [121]. In mice with diabetes mellitus, ipragliflozin improves endothelial function by phosphorylating eNOS (Ser1177), increasing eNOS activity, and restoring Akt phosphorylation. Additionally, it decreases the expression of inflammatory molecules, such as ICAM-1, VCAM-1, and MCP-1, in the abdominal aorta [122].

### 5.3. Sodium–glucose Cotransporter 2 Inhibitors (SGLT2i) as an Emerging Treatment for Renal Lipotoxicity

SGLT2i demonstrate promising effects in renal lipotoxicity. For instance, dapagliflozin reduces podocyte cholesterol accumulation by normalizing free and total cholesterol levels and increasing the expression of podocin, nephrin, and ABCA1, mediated by Krüppel-like factor 5 (KLF-5) (Figure 4). Additionally, dapagliflozin reduces the expression of apoptotic markers, such as Bax and caspase 3, while the anti-apoptotic protein Bcl-2 was upregulated (Figure 4) [123].

The inhibition of SGLT2 activity decreases systolic blood pressure, kidney weight/body weight ratio, urinary albumin, and lipid accumulation in the kidney via inhibition of ChERBP-β, pyruvate kinase L, SCD1, and DGAT1, key transcriptional factors and enzymes involved in fatty acid and triglyceride synthesis. It also induces anti-inflammatory effects by inhibiting macrophage accumulation and expression of CD68, NF-kB p65, Toll-like receptor 4 (TLR4), MCP-1, and osteopontin. The beneficial effects are also associated with reduced mesangial expansion and extracellular matrix proteins, including fibronectin and type IV collagen. In contrast, podocyte markers, such as Wnt family member 1 (WT1) and synaptopodin, are preserved [124].

Empagliflozin downregulates CD36 via PPAR-γ, ameliorating palmitate-induced inflammation and lipotoxicity in renal proximal tubular cells [125]. Dapagliflozin treatment enhances expressions of PGC-1α and PPARα while suppressing expressions of fatty acid synthase, SREBP1, and CD36 (Figure 4) [126].

Diabetes reprograms the metabolic profile in the proximal tubule by switching from fatty acid utilization to glycolysis, leading to lipid accumulation and increased expression of HIF-1α. SGLT2 inhibitors rectify diabetes-induced metabolic reprogramming in proximal tubular epithelial cells by inhibiting HIF-1α expression [127]. Empagliflozin improves mitochondrial dynamics, biogenesis, and functions and regulates protein expression, such as mTOR, raptor, and ULK1 activity, resulting in increased autophagy activity [128]. Additionally, SGLT2 inhibitors reduce OS by improving mitochondrial function and increasing the rate of fatty acid oxidation and AMPK activity by phosphorylating the threonine-172 (Thr^172^) residue [129]. Canagliflozin inhibits complex I of the respiratory chain, potentially mediated by the PPARα receptor. This action prevents the re-oxidation of nicotinamide adenine dinucleotide (NADH) and flavin adenine dinucleotide (FADH) generated by fat oxidation, thereby activating AMPK [129,130]. Other reports have described that SGLT2i improved mitochondrial function, biogenesis, mitophagy, and β-oxidation through modulation of gene expression. However, the mechanistic studies focusing on kidney function, MetS, and patients are limited [131].

## 6. Discussion

MetS encompasses a constellation of disorders that lead to increased lipid synthesis and accumulation in peripheral tissues, including the kidney. While lipids play essential roles as structural components and regulators in various cellular functions, excessive intake or disruptions in their metabolism can lead to diseases such as atherosclerosis and CKD. In patients, CKD is characterized by elevated levels of TG, very-low-density lipoprotein (VLDL), intermediate-density lipoprotein (IDL), small dense LDL (sdLDL), and chylomicron remnants [3,43]. Dyslipidemia, a prominent feature of MetS, results in excessive lipid accumulation within the kidneys at both the glomerular and tubular levels. This lipid accumulation triggers a cascade of detrimental effects, including mitochondrial dysfunction, oxidative stress, actin cytoskeleton remodeling, IR, activation of the RAAS, and inflammatory responses and ultimately culminates in structural damage and impaired kidney function [3,40].

Studies have reported overexpression of CD36 and reduced β-oxidation, both associated with lipid droplet accumulation in the kidney, contributing to kidney disease [53]. Additionally, JAML expression has been linked to serum creatinine levels and lipid accumulation through the SIRT1–AMPK–SREBP1 signaling pathway [57]. It has been described that SIRT1 inhibits the activity of SREBP1 and its target genes, such as PPARs and ChREBP, but is downregulated by JAML [55].

Further, mitochondrial damage caused by imbalances in enzyme expression, such as ACC, ACS, malonyl-CoA, CPT-1, and CPT2, disrupts β-oxidation, leading to an increase in acetyl-CoA, a limiting substrate of lipogenesis, resulting in lipid accumulation [132,133].

Pharmacological therapy for MetS primarily aims to control RFs to prevent cardiovascular complications, although strict management of these factors is often lacking in clinical practice. The mortality rate increases with the number of RFs, underscoring the importance of comprehensive management [3,8,134]. Achieving composite goals in lipids, blood pressure, and glucose has been demonstrated to reduce the risk for recurrent major adverse cardiovascular events (MACE) by 80% [135]. In this context, SGLT2 inhibitors, initially developed as antidiabetic drugs, have demonstrated pleiotropic effects, beneficial for MetS. These include metabolic, antioxidant, anti-inflammatory, and antifibrotic effects across multiple organs, including the kidney.

On the other hand, despite the simultaneous use of multiple drugs, including diuretics, hypertension remains one of the leading causes of death worldwide [136]. In this regard, the use of SGLT2i has demonstrated antihypertensive effects, which may be attributed to their impact on obesity, insulin resistance, and natriuretic actions [113,130,137,138]. These antihypertensive effects can extend to the kidneys and heart, offering protection against the harmful consequences of hypertension and preserving organ function.

Notably, SGLT2i have shown promise in improving mitochondrial function [139]. Therefore, SGLT2 inhibition ameliorates metabolic disorder and obesity-induced cardiomyocyte injury and mitochondrial remodeling by reducing lipotoxicity, mitochondrial ROS production, mitochondrial calcium (Ca^2+^) overload, and the levels of associated proteins, such as superoxide dismutase 1 (SOD1), as well as by the downregulation of mitofusin 2 (mfn2), SIRT1, and SERCA [140].

In addition, combination therapy with SGLT2i alongside other antidiabetic medications, such as GLP-1 receptor agonists (GLP-1 RA) or dipeptidyl peptidase 4 inhibitors (DPP4I), has yielded promising results in improving glycemic control, lipid profiles, and blood pressure [141,142,143].

Clinical evidence suggests that SGLT2 inhibitors offer a potential therapeutic solution for CKD and MetS. They have demonstrated benefits in glycemic regulation, which may help increase insulin sensitivity. Also, SGLT2i can enhance lipid metabolism, mitochondrial function, and antioxidant capacity, improving the lipid profile, which is essential for preserving kidney function and reducing kidney damage biomarkers.

The evidence presented suggests that empagliflozin, an SGLT2 inhibitor, may be a viable treatment option for the interconnected factors contributing to metabolic syndrome and its associated complications, particularly CKD. Empagliflozin’s beneficial effects on glycemic control, blood pressure regulation, weight management, and renal function support its potential as a multi-faceted therapeutic agent for this complex condition. However, it is important to acknowledge the limitations of the available data. The absence of comprehensive data on the long-term effects of empagliflozin on metabolic syndrome coupled with the reliance on preclinical studies and small-sample clinical trials necessitate further research to understand its therapeutic potential fully. Additionally, the potential for synergistic interactions between empagliflozin and other drugs, particularly metformin, warrants further investigation.

Future research should prioritize conducting large-scale, long-term clinical trials to evaluate the safety and efficacy of SGLT2i in diverse populations with metabolic syndrome. Additionally, population-based and mechanistic studies in humans are needed to elucidate the precise mechanisms by which empagliflozin exerts its beneficial effects and to identify potential biomarkers of response.

Overall, evidence suggests the potential of SGLT2 inhibitors in managing RFs associated with MetS and protecting against kidney damage. However, further studies are warranted to explore their full therapeutic potential in this context.

## 7. Conclusions

MetS encompasses a cluster of interconnected RFs, including hyperglycemia, obesity, dyslipidemia, IR, and hypertension, which collectively predispose individuals to CKD. In this context, SGLT2i, through their modulation of cellular mechanisms, have demonstrated efficacy in improving lipid metabolism, ameliorating dyslipidemia, and enhancing insulin sensitivity. These effects collectively attenuate lipid accumulation and toxicity, which are key drivers of renal dysfunction in MetS. The potential of SGLT2i to mitigate metabolic and hemodynamic disturbances underscores their therapeutic promise in addressing the complex pathophysiology of MetS and its associated renal complications.

Compelling evidence from clinical and preclinical investigations supports the use of SGLT2i, either as monotherapy or in combination with other antidiabetic agents, as an attractive therapeutic option for managing MetS. Their pleiotropic effects, extending beyond glycemic control, highlight their potential to target multiple facets of MetS, ultimately reducing the risk of CKD and improving patient outcomes. Further research is warranted to optimize treatment regimens and explore the full potential of SGLT2i in the comprehensive management of MetS.

## Figures and Tables

**Table 1:** Criteria for metabolic syndrome diagnosis, according to different health organizations.

Organization	NCEP ATPIII 2005	IDF 2005	WHO 1998
Criteria	Any of three or more of the following five components	Central obesity plus any two other factors	IGT, IFG, T2DM, or reduced insulin sensitivity plus any two of the following
Obesity	WC ≥ 102 cm in men or ≥88 cm in women population-specific increased WC cutoffs		Men: WHR > 0.9 Women: WHR > 0.85 and/or BMI > 30 kg/m^2^
Blood Pressure	≥130/85 mmHg or on antihypertensive therapy	≥130/85 mmHg or on antihypertensive therapy	≥140/90 mmHg
Lipid profile	TG ≥ 150 mg/dL or on therapy lowering TG	TG ≥ 150 mg/dL or chronic treatment for lipid abnormality and reduced HDL-c: < 40 mg/dL for males and < 50 mg/dL for females	TG ≥ 150 mg/dL
	HDL-c < 40 mg/dL in men or HDL-c < 50 mg/dL in women on therapy increasing HDL-c		
Glucose	≥100 mg/dL (including T2DM)	≥100 mg/dL (including T2DM)	IGT, IFG, or T2DM
Microalbuminuria	Urinary excretion rate > 20 mg/min or		
	Albumin/creatinine >30 mg/min		

Body mass index (BMI), impaired glucose tolerance (IGT), impaired fasting glucose (IFG), type 2 diabetes mellitus (T2DM), waist circumference (WC), waist/hip circumference ratio (WHR), triglycerides (TG), high-density lipoprotein (HDL) cholesterol (HDL-c), World Health Organization 1998 (WHO, 1998), National Cholesterol Education Program Adult Treatment Panel III (ATP III), and the International Diabetes Federation (IDF, 2005), adapted from Xu et al. and Bovolini et al. [2,4].

## Data Availability

Not applicable.

## References

[1] Rizvi A.A., Stoian A.P., Rizzo M. (2021). Metabolic Syndrome: From Molecular Mechanisms to Novel Therapies. Int. J. Mol. Sci..

[2] Bovolini A., Garcia J., Andrade M.A., Duarte J.A. (2021). Metabolic Syndrome Pathophysiology and Predisposing Factors. Int. J. Sports Med..

[3] Arellano Buendia A.S., Juárez Rojas J.G., García-Arroyo F., Aparicio Trejo O.E., Sánchez-Muñoz F., Argüello-García R., Sánchez-Lozada L.G., Bojalil R., Osorio-Alonso H. (2023). Antioxidant and anti-inflammatory effects of allicin in the kidney of an experimental model of metabolic syndrome. PeerJ.

[4] Xu H., Li X., Adams H., Kubena K., Guo S. (2018). Etiology of Metabolic Syndrome and Dietary Intervention. Int. J. Mol. Sci..

[5] Noubiap J.J., Nansseu J.R., Lontchi-Yimagou E., Nkeck J.R., Nyaga U.F., Ngouo A.T., Tounouga D.N., Tianyi F.L., Foka A.J., Ndoadoumgue A.L. (2022). Geographic distribution of metabolic syndrome and its components in the general adult population: A meta-analysis of global data from 28 million individuals. Diabetes Res. Clin. Pract..

[6] Aguilar M., Bhuket T., Torres S., Liu B., Wong R.J. (2015). Prevalence of the metabolic syndrome in the United States, 2003–2012. Jama.

[7] Rojas-Martínez R., Aguilar-Salinas C.A., Romero-Martínez M., Castro-Porras L., Gómez-Velasco D., Mehta R. (2021). Trends in the prevalence of metabolic syndrome and its components in Mexican adults, 2006-2018. Salud Publica Mex.

[8] Li W., Chen D., Peng Y., Lu Z., Kwan M.P., Tse L.A. (2023). Association Between Metabolic Syndrome and Mortality: Prospective Cohort Study. JMIR Public. Health Surveill..

[9] Campos-Nonato I., Hernández-Barrera L., Pedroza-Tobías A., Medina C., Barquera S. (2018). Hypertension in Mexican adults: Prevalence, diagnosis and type of treatment. Ensanut MC 2016. Salud Publica Mex..

[10] Wahl P., Ducasa G.M., Fornoni A. (2016). Systemic and renal lipids in kidney disease development and progression. Am. J. Physiol. Renal Physiol..

[11] Zhang X., Lerman L.O. (2017). The metabolic syndrome and chronic kidney disease. Transl. Res..

[12] De Lorenzo A., Gratteri S., Gualtieri P., Cammarano A., Bertucci P., Di Renzo L. (2019). Why primary obesity is a disease?. J. Transl. Med..

[13] Neves R.O., Rocha A.D.S., de Vargas B.O., Kretzer D.C., de Matos S., Goldani M.Z., von Diemen L., Magalhães J.A.A., Bernardi J.R. (2023). Obesity Cut-Off Points Using Prepregnancy Body Mass Index according to Cardiometabolic Conditions in Pregnancy. J. Pregnancy.

[14] Barquera S., Hernández-Barrera L., Trejo-Valdivia B., Shamah T., Campos-Nonato I., Rivera-Dommarco J. (2020). Obesity in Mexico, prevalence andtrends in adults. Ensanut 2018-19. Salud Publica Mex..

[15] Koenen M., Hill M.A., Cohen P., Sowers J.R. (2021). Obesity, Adipose Tissue and Vascular Dysfunction. Circ. Res..

[16] Unamuno X., Gómez-Ambrosi J., Rodríguez A., Becerril S., Frühbeck G., Catalán V. (2018). Adipokine dysregulation and adipose tissue inflammation in human obesity. Eur. J. Clin. Investig..

[17] Sakers A., De Siqueira M.K., Seale P., Villanueva C.J. (2022). Adipose-tissue plasticity in health and disease. Cell.

[18] Ferrara D., Montecucco F., Dallegri F., Carbone F. (2019). Impact of different ectopic fat depots on cardiovascular and metabolic diseases. J. Cell Physiol..

[19] Pereira S., Cline D.L., Glavas M.M., Covey S.D., Kieffer T.J. (2021). Tissue-Specific Effects of Leptin on Glucose and Lipid Metabolism. Endocr. Rev..

[20] Stenkula K.G., Erlanson-Albertsson C. (2018). Adipose cell size: Importance in health and disease. Am. J. Physiol. Regul. Integr. Comp. Physiol..

[21] Vekic J., Stefanovic A., Zeljkovic A. (2023). Obesity and Dyslipidemia: A Review of Current Evidence. Curr. Obes. Rep..

[22] Ruiz-García A., Arranz-Martínez E., López-Uriarte B., Rivera-Teijido M., Palacios-Martínez D., Dávila-Blázquez G.M., Rosillo-González A., González-Posada Delgado J.A., Mariño-Suárez J.E., Revilla-Pascual E. (2020). Prevalence of hypertriglyceridemia in adults and related cardiometabolic factors. SIMETAP-HTG study. Clin. Investig. Arterioscler..

[23] Ali N., Samadder M., Shourove J.H., Taher A., Islam F. (2023). Prevalence and factors associated with metabolic syndrome in university students and academic staff in Bangladesh. Sci. Rep..

[24] Márquez A.B., Nazir S., van der Vorst E.P.C. (2020). High-Density Lipoprotein Modifications: A Pathological Consequence or Cause of Disease Progression?. Biomedicines.

[25] Denimal D. (2024). Antioxidant and Anti-Inflammatory Functions of High-Density Lipoprotein in Type 1 and Type 2 Diabetes. Antioxidants.

[26] Mineo C., Yuhanna I.S., Quon M.J., Shaul P.W. (2003). High density lipoprotein-induced endothelial nitric-oxide synthase activation is mediated by Akt and MAP kinases. J. Biol. Chem..

[27] Khatana C., Saini N.K., Chakrabarti S., Saini V., Sharma A., Saini R.V., Saini A.K. (2020). Mechanistic Insights into the Oxidized Low-Density Lipoprotein-Induced Atherosclerosis. Oxid. Med. Cell Longev..

[28] Noels H., Lehrke M., Vanholder R., Jankowski J. (2021). Lipoproteins and fatty acids in chronic kidney disease: Molecular and metabolic alterations. Nat. Rev. Nephrol..

[29] Dincer N., Dagel T., Afsar B., Covic A., Ortiz A., Kanbay M. (2019). The effect of chronic kidney disease on lipid metabolism. Int. Urol. Nephrol..

[30] Cignarelli A., Genchi V.A., Perrini S., Natalicchio A., Laviola L., Giorgino F. (2019). Insulin and Insulin Receptors in Adipose Tissue Development. Int. J. Mol. Sci..

[31] Thongnak L., Pongchaidecha A., Lungkaphin A. (2020). Renal Lipid Metabolism and Lipotoxicity in Diabetes. Am. J. Med. Sci..

[32] Fahed G., Aoun L., Bou Zerdan M., Allam S., Bou Zerdan M., Bouferraa Y., Assi H.I. (2022). Metabolic Syndrome: Updates on Pathophysiology and Management in 2021. Int. J. Mol. Sci..

[33] Pantoja-Torres B., Toro-Huamanchumo C.J., Urrunaga-Pastor D., Guarnizo-Poma M., Lazaro-Alcantara H., Paico-Palacios S., Del Carmen Ranilla-Seguin V., Benites-Zapata V.A. (2019). High triglycerides to HDL-cholesterol ratio is associated with insulin resistance in normal-weight healthy adults. Diabetes Metab. Syndr..

[34] Lee S.H., Park S.Y., Choi C.S. (2022). Insulin Resistance: From Mechanisms to Therapeutic Strategies. Diabetes Metab. J..

[35] Reaven G.M., Greenfield M.S. (1981). Diabetic hypertriglyceridemia: Evidence for three clinical syndromes. Diabetes.

[36] Afshinnia F., Nair V., Lin J., Rajendiran T.M., Soni T., Byun J., Sharma K., Fort P.E., Gardner T.W., Looker H.C. (2019). Increased lipogenesis and impaired β-oxidation predict type 2 diabetic kidney disease progression in American Indians. JCI Insight.

[37] da Silva A.A., do Carmo J.M., Li X., Wang Z., Mouton A.J., Hall J.E. (2020). Role of Hyperinsulinemia and Insulin Resistance in Hypertension: Metabolic Syndrome Revisited. Can. J. Cardiol..

[38] García-Carrasco A., Izquierdo-Lahuerta A., Medina-Gómez G. (2021). The Kidney-Heart Connection in Obesity. Nephron.

[39] Whaley-Connell A., Sowers J.R. (2017). Insulin Resistance in Kidney Disease: Is There a Distinct Role Separate from That of Diabetes or Obesity?. Cardiorenal Med..

[40] Opazo-Ríos L., Mas S., Marín-Royo G., Mezzano S., Gómez-Guerrero C., Moreno J.A., Egido J. (2020). Lipotoxicity and Diabetic Nephropathy: Novel Mechanistic Insights and Therapeutic Opportunities. Int. J. Mol. Sci..

[41] Hall J.E., Mouton A.J., da Silva A.A., Omoto A.C.M., Wang Z., Li X., do Carmo J.M. (2021). Obesity, kidney dysfunction, and inflammation: Interactions in hypertension. Cardiovasc. Res..

[42] Wei P.Z., Szeto C.C. (2019). Mitochondrial dysfunction in diabetic kidney disease. Clin. Chim. Acta.

[43] Miricescu D., Balan D.G., Tulin A., Stiru O., Vacaroiu I.A., Mihai D.A., Popa C.C., Enyedi M., Nedelea A.S., Nica A.E. (2021). Impact of adipose tissue in chronic kidney disease development (Review). Exp. Ther. Med..

[44] Yim H.E., Yoo K.H. (2021). Obesity and chronic kidney disease: Prevalence, mechanism, and management. Clin. Exp. Pediatr..

[45] Kon V., Yang H.C., Smith L.E., Vickers K.C., Linton M.F. (2021). High-Density Lipoproteins in Kidney Disease. Int. J. Mol. Sci..

[46] Strazzella A., Ossoli A., Calabresi L. (2021). High-Density Lipoproteins and the Kidney. Cells.

[47] Stadler J.T., Marsche G. (2020). Obesity-Related Changes in High-Density Lipoprotein Metabolism and Function. Int. J. Mol. Sci..

[48] Miljkovic M., Stefanovic A., Simic-Ogrizovic S., Vekic J., Bogavac-Stanojevic N., Cerne D., Kocbek P., Marc J., Jelic-Ivanovic Z., Spasojevic-Kalimanovska V. (2018). Association of Dyslipidemia, Oxidative Stress, and Inflammation With Redox Status in VLDL, LDL, and HDL Lipoproteins in Patients With Renal Disease. Angiology.

[49] Wang A., Lin Y., Liang B., Zhao X., Qiu M., Huang H., Li C., Wang W., Kong Y. (2022). Statins attenuate cholesterol-induced ROS via inhibiting NOX2/NOX4 and mitochondrial pathway in collecting ducts of the kidney. BMC Nephrol..

[50] Rogacka D. (2021). Insulin resistance in glomerular podocytes: Potential mechanisms of induction. Arch. Biochem. Biophys..

[51] Merscher S., Pedigo C.E., Mendez A.J. (2014). Metabolism, energetics, and lipid biology in the podocyte—Cellular cholesterol-mediated glomerular injury. Front. Endocrinol..

[52] Wang H., Zhang S., Guo J. (2021). Lipotoxic Proximal Tubular Injury: A Primary Event in Diabetic Kidney Disease. Front. Med..

[53] Yang X., Okamura D.M., Lu X., Chen Y., Moorhead J., Varghese Z., Ruan X.Z. (2017). CD36 in chronic kidney disease: Novel insights and therapeutic opportunities. Nat. Rev. Nephrol..

[54] Kim J.J., Wilbon S.S., Fornoni A. (2021). Podocyte Lipotoxicity in CKD. Kidney360.

[55] Sun Y., Cui S., Hou Y., Yi F. (2021). The Updates of Podocyte Lipid Metabolism in Proteinuric Kidney Disease. Kidney Dis..

[56] Ebnet K. (2017). Junctional Adhesion Molecules (JAMs): Cell Adhesion Receptors With Pleiotropic Functions in Cell Physiology and Development. Physiol. Rev..

[57] Fu Y., Sun Y., Wang M., Hou Y., Huang W., Zhou D., Wang Z., Yang S., Tang W., Zhen J. (2020). Elevation of JAML Promotes Diabetic Kidney Disease by Modulating Podocyte Lipid Metabolism. Cell Metab..

[58] Zhang R., Zhang K. (2024). A unified model for regulating lipoprotein lipase activity. Trends Endocrinol. Metab..

[59] Sylvers-Davie K.L., Davies B.S.J. (2021). Regulation of lipoprotein metabolism by ANGPTL3, ANGPTL4, and ANGPTL8. Am. J. Physiol. Endocrinol. Metab..

[60] Clement L.C., Macé C., Avila-Casado C., Joles J.A., Kersten S., Chugh S.S. (2014). Circulating angiopoietin-like 4 links proteinuria with hypertriglyceridemia in nephrotic syndrome. Nat. Med..

[61] Aryal B., Singh A.K., Zhang X., Varela L., Rotllan N., Goedeke L., Chaube B., Camporez J.P., Vatner D.F., Horvath T.L. (2018). Absence of ANGPTL4 in adipose tissue improves glucose tolerance and attenuates atherogenesis. JCI Insight.

[62] Li G., Lu D., Wang J., Yue S., Tan M., Liu M., Gao X. (2022). ANGPTL3 is involved in kidney injury in high-fat diet-fed mice by suppressing ACTN4 expression. Lipids Health Dis..

[63] Li Y., Gong W., Liu J., Chen X., Suo Y., Yang H., Gao X. (2022). Angiopoietin-like protein 4 promotes hyperlipidemia-induced renal injury by down-regulating the expression of ACTN4. Biochem. Biophys. Res. Commun..

[64] Qiu W., Huang L., Li Y., Liu Q., Lv Y. (2023). Dysregulation of Angiopoietin-like-4 Associated with Hyperlipidemia-induced Renal Injury by AMPK/ACC Pathway. Curr. Pharm. Des..

[65] Li L., Yang J., Li F., Gao F., Zhu L., Hao J. (2021). FBXW7 mediates high glucose-induced SREBP-1 expression in renal tubular cells of diabetic nephropathy under PI3K/Akt pathway regulation. Mol. Med. Rep..

[66] Jun H., Song Z., Chen W., Zanhua R., Yonghong S., Shuxia L., Huijun D. (2009). In vivo and in vitro effects of SREBP-1 on diabetic renal tubular lipid accumulation and RNAi-mediated gene silencing study. Histochem. Cell Biol..

[67] Li L., Zhao Z., Xia J., Xin L., Chen Y., Yang S., Li K. (2015). A Long-Term High-Fat/High-Sucrose Diet Promotes Kidney Lipid Deposition and Causes Apoptosis and Glomerular Hypertrophy in Bama Minipigs. PLoS ONE.

[68] Tominaga T., Dutta R.K., Joladarashi D., Doi T., Reddy J.K., Kanwar Y.S. (2016). Transcriptional and Translational Modulation of myo-Inositol Oxygenase (Miox) by Fatty Acids: Implications in Renal Tubular Injury Induced in Obesity and Diabetes. J. Biol. Chem..

[69] Lu J., Li X.Q., Chen P.P., Zhang J.X., Li L., Wang G.H., Liu X.Q., Jiang C.M., Ma K.L. (2024). Acetyl-CoA synthetase 2 promotes diabetic renal tubular injury in mice by rewiring fatty acid metabolism through SIRT1/ChREBP pathway. Acta Pharmacol. Sin..

[70] Katz L.S., Xu S., Ge K., Scott D.K., Gershengorn M.C. (2018). T3 and Glucose Coordinately Stimulate ChREBP-Mediated Ucp1 Expression in Brown Adipocytes From Male Mice. Endocrinology.

[71] Jin K., Norris K., Vaziri N.D. (2013). Dysregulation of hepatic fatty acid metabolism in chronic kidney disease. Nephrol. Dial. Transplant..

[72] Chen N., Mu L., Yang Z., Du C., Wu M., Song S., Yuan C., Shi Y. (2021). Carbohydrate response element-binding protein regulates lipid metabolism via mTOR complex1 in diabetic nephropathy. J. Cell Physiol..

[73] Lin T.A., Wu V.C., Wang C.Y. (2019). Autophagy in Chronic Kidney Diseases. Cells.

[74] Li Z., Li J., Miao X., Cui W., Miao L., Cai L. (2021). A minireview: Role of AMP-activated protein kinase (AMPK) signaling in obesity-related renal injury. Life Sci..

[75] Juszczak F., Vlassembrouck M., Botton O., Zwakhals T., Decarnoncle M., Tassin A., Caron N., Declèves A.-E. (2021). Delayed Exercise Training Improves Obesity-Induced Chronic Kidney Disease by Activating AMPK Pathway in High-Fat Diet-Fed Mice. Int. J. Mol. Sci..

[76] Lee E.S., Kwon M.H., Kim H.M., Kim N., Kim Y.M., Kim H.S., Lee E.Y., Chung C.H. (2019). Dibenzoylmethane ameliorates lipid-induced inflammation and oxidative injury in diabetic nephropathy. J. Endocrinol..

[77] Zhou Y., Chen Z., Zhou H., Niu B., Liu J., Li Y., Mi Y., Li P. (2023). ACT001 Alleviates chronic kidney injury induced by a high-fat diet in mice through the GPR43/AMPK pathway. Lipids Health Dis..

[78] Corrales P., Vidal-Puig A., Medina-Gómez G. (2018). PPARs and Metabolic Disorders Associated with Challenged Adipose Tissue Plasticity. Int. J. Mol. Sci..

[79] Libby A.E., Jones B., Lopez-Santiago I., Rowland E., Levi M. (2021). Nuclear receptors in the kidney during health and disease. Mol. Aspects Med..

[80] Corrales P., Izquierdo-Lahuerta A., Medina-Gómez G. (2018). Maintenance of Kidney Metabolic Homeostasis by PPAR Gamma. Int. J. Mol. Sci..

[81] Gao J., Gu Z. (2022). The Role of Peroxisome Proliferator-Activated Receptors in Kidney Diseases. Front. Pharmacol..

[82] Yuan Q., Tang B., Zhang C. (2022). Signaling pathways of chronic kidney diseases, implications for therapeutics. Signal Transduct. Target. Ther..

[83] Longo N., Frigeni M., Pasquali M. (2016). Carnitine transport and fatty acid oxidation. Biochim. Biophys. Acta.

[84] Console L., Scalise M., Giangregorio N., Tonazzi A., Barile M., Indiveri C. (2020). The Link Between the Mitochondrial Fatty Acid Oxidation Derangement and Kidney Injury. Front. Physiol..

[85] Lee M., Katerelos M., Gleich K., Galic S., Kemp B.E., Mount P.F., Power D.A. (2018). Phosphorylation of Acetyl-CoA Carboxylase by AMPK Reduces Renal Fibrosis and Is Essential for the Anti-Fibrotic Effect of Metformin. J. Am. Soc. Nephrol..

[86] Clark A.J., Parikh S.M. (2021). Targeting energy pathways in kidney disease: The roles of sirtuins, AMPK, and PGC1α. Kidney Int..

[87] Hou Y., Tan E., Shi H., Ren X., Wan X., Wu W., Chen Y., Niu H., Zhu G., Li J. (2024). Mitochondrial oxidative damage reprograms lipid metabolism of renal tubular epithelial cells in the diabetic kidney. Cell Mol. Life Sci..

[88] Ceja-Galicia Z.A., García-Arroyo F.E., Aparicio-Trejo O.E., El-Hafidi M., Gonzaga-Sánchez G., León-Contreras J.C., Hernández-Pando R., Guevara-Cruz M., Tovar A.R., Rojas-Morales P. (2022). Therapeutic Effect of Curcumin on 5/6Nx Hypertriglyceridemia: Association with the Improvement of Renal Mitochondrial β-Oxidation and Lipid Metabolism in Kidney and Liver. Antioxidants.

[89] Li S.Y., Susztak K. (2018). The Role of Peroxisome Proliferator-Activated Receptor γ Coactivator 1α (PGC-1α) in Kidney Disease. Semin. Nephrol..

[90] Jang S., Jang B.H., Ko Y., Sasaki Y., Park J.S., Hwang E.H., Song Y.K., Shin Y.C., Ko S.G. (2016). Herbal Medicines for Treating Metabolic Syndrome: A Systematic Review of Randomized Controlled Trials. Evid. Based Complement. Alternat Med..

[91] Ahmed M., Kumari N., Mirgani Z., Saeed A., Ramadan A., Ahmed M.H., Almobarak A.O. (2022). Metabolic syndrome; Definition, Pathogenesis, Elements, and the Effects of medicinal plants on it’s elements. J. Diabetes Metab. Disord..

[92] Noce A., Di Lauro M., Di Daniele F., Pietroboni Zaitseva A., Marrone G., Borboni P., Di Daniele N. (2021). Natural Bioactive Compounds Useful in Clinical Management of Metabolic Syndrome. Nutrients.

[93] Chaachouay N., Zidane L. (2024). Plant-Derived Natural Products: A Source for Drug Discovery and Development. Drugs Drug Candidates.

[94] Qu Y., Chan J.Y., Wong C.W., Cheng L., Xu C., Leung A.W., Lau C.B. (2015). Antidiabetic Effect of Schisandrae Chinensis Fructus Involves Inhibition of the Sodium Glucose Cotransporter. Drug Dev. Res..

[95] Liu H., Wu C., Wang S., Gao S., Liu J., Dong Z., Zhang B., Liu M., Sun X., Guo P. (2015). Extracts and lignans of Schisandra chinensis fruit alter lipid and glucose metabolism in vivo and in vitro. J. Funct. Foods.

[96] Sato S., Takeo J., Aoyama C., Kawahara H. (2007). Na^+^-glucose cotransporter (SGLT) inhibitory flavonoids from the roots of Sophora flavescens. Bioorg Med. Chem..

[97] Yang X., Yang J., Xu C., Huang M., Zhou Q., Lv J., Ma X., Ke C., Ye Y., Shu G. (2015). Antidiabetic effects of flavonoids from Sophora flavescens EtOAc extract in type 2 diabetic KK-ay mice. J. Ethnopharmacol..

[98] Yang Y., Liu Y., Gao Y., Zhao K., Li Z., Luo Y., Chen L. (2022). Exploring the anti-diabetic effects and the underlying mechanisms of ethyl acetate extract from Sophora flavescens by integrating network pharmacology and pharmacological evaluation. Tradit. Med. Res..

[99] Moradi-Marjaneh R., Paseban M., Sahebkar A. (2019). Natural products with SGLT2 inhibitory activity: Possibilities of application for the treatment of diabetes. Phytother. Res..

[100] Shimokawa Y., Akao Y., Hirasawa Y., Awang K., Hadi A.H., Sato S., Aoyama C., Takeo J., Shiro M., Morita H. (2010). Gneyulins A and B, stilbene trimers, and noidesols A and B, dihydroflavonol-C-glucosides, from the bark of Gnetum gnemonoides. J. Nat. Prod..

[101] Morita H., Deguchi J., Motegi Y., Sato S., Aoyama C., Takeo J., Shiro M., Hirasawa Y. (2010). Cyclic diarylheptanoids as Na+-glucose cotransporter (SGLT) inhibitors from Acer nikoense. Bioorganic Med. Chem. Lett..

[102] Arai H., Hirasawa Y., Rahman A., Kusumawati I., Zaini N.C., Sato S., Aoyama C., Takeo J., Morita H. (2010). Alstiphyllanines E-H, picraline and ajmaline-type alkaloids from Alstonia macrophylla inhibiting sodium glucose cotransporter. Bioorg. Med. Chem..

[103] Khanam S., Mishra D.A., Shahid A., Pujari N.M. (2022). Therapeutic indication of Phloridzin: A new Gleam for metabolic disorders. Phytomed. Plus.

[104] Maisto M., Piccolo V., Novellino E., Schiano E., Iannuzzo F., Ciampaglia R., Summa V., Tenore G.C. (2022). Optimization of Phlorizin Extraction from Annurca Apple Tree Leaves Using Response Surface Methodology. Antioxidants.

[105] Osorio H., Coronel I., Arellano A., Pacheco U., Bautista R., Franco M., Escalante B. (2012). Sodium-glucose cotransporter inhibition prevents oxidative stress in the kidney of diabetic rats. Oxid. Med. Cell Longev..

[106] Osorio H., Bautista R., Rios A., Franco M., Arellano A., Vargas-Robles H., Romo E., Escalante B. (2010). Effect of phlorizin on SGLT2 expression in the kidney of diabetic rats. J. Nephrol..

[107] Mei X., Zhang X., Wang Z., Gao Z., Liu G., Hu H., Zou L., Li X. (2016). Insulin Sensitivity-Enhancing Activity of Phlorizin Is Associated with Lipopolysaccharide Decrease and Gut Microbiota Changes in Obese and Type 2 Diabetes (db/db) Mice. J. Agric. Food Chem..

[108] Castañeda A.M., Dutra-Rufato A., Juarez M.J., Grosembacher L., Gonzalez-Torres H., Musso C.G. (2021). Sodium-glucose cotransporter 2 inhibitors (SGLT2i): Renal implications. Int. Urol. Nephrol..

[109] Pedraza-Chaverri J., Sánchez-Lozada L.G., Osorio-Alonso H., Tapia E., Scholze A. (2016). New Pathogenic Concepts and Therapeutic Approaches to Oxidative Stress in Chronic Kidney Disease. Oxid. Med. Cell Longev..

[110] Andreea M.M., Surabhi S., Razvan-Ionut P., Lucia C., Camelia N., Emil T., Tiberiu N.I. (2023). Sodium-Glucose Cotransporter 2 (SGLT2) Inhibitors: Harms or Unexpected Benefits?. Medicina.

[111] Zhang J., Huan Y., Leibensperger M., Seo B., Song Y. (2022). Comparative Effects of Sodium-Glucose Cotransporter 2 Inhibitors on Serum Electrolyte Levels in Patients with Type 2 Diabetes: A Pairwise and Network Meta-Analysis of Randomized Controlled Trials. Kidney360.

[112] Zhang Q., Zhou S., Liu L. (2023). Efficacy and safety evaluation of SGLT2i on blood pressure control in patients with type 2 diabetes and hypertension: A new meta-analysis. Diabetol. Metab. Syndr..

[113] Ni L., Yuan C., Chen G., Zhang C., Wu X. (2020). SGLT2i: Beyond the glucose-lowering effect. Cardiovasc. Diabetol..

[114] Chino Y., Samukawa Y., Sakai S., Nakai Y., Yamaguchi J., Nakanishi T., Tamai I. (2014). SGLT2 inhibitor lowers serum uric acid through alteration of uric acid transport activity in renal tubule by increased glycosuria. Biopharm. Drug Dispos..

[115] Sánchez-Briales P., Marques Vidas M., López-Sánchez P., López-Illázquez M.V., Martín-Testillano L., Vedat-Ali A., Portolés J. (2024). The Uricosuric Effect of SGLT2 Inhibitors Is Maintained in the Long Term in Patients with Chronic Kidney Disease and Type 2 Diabetes Mellitus. J. Clin. Med..

[116] Somagutta M.K.R., Luvsannyam E., Jain M., Cuddapah G.V., Pelluru S., Mustafa N., Nasereldin D.S., Pendyala S.K., Jarapala N., Padamati B. (2022). Sodium glucose co-transport 2 inhibitors for gout treatment. Discoveries.

[117] Szekeres Z., Sandor B., Bognar Z., Ramadan F.H.J., Palfi A., Bodis B., Toth K., Szabados E. (2023). Clinical Study of Metabolic Parameters, Leptin and the SGLT2 Inhibitor Empagliflozin among Patients with Obesity and Type 2 Diabetes. Int. J. Mol. Sci..

[118] Kohlmorgen C., Gerfer S., Feldmann K., Twarock S., Hartwig S., Lehr S., Klier M., Krüger I., Helten C., Keul P. (2021). Dapagliflozin reduces thrombin generation and platelet activation: Implications for cardiovascular risk reduction in type 2 diabetes mellitus. Diabetologia.

[119] Xu L., Nagata N., Nagashimada M., Zhuge F., Ni Y., Chen G., Mayoux E., Kaneko S., Ota T. (2017). SGLT2 Inhibition by Empagliflozin Promotes Fat Utilization and Browning and Attenuates Inflammation and Insulin Resistance by Polarizing M2 Macrophages in Diet-induced Obese Mice. EBioMedicine.

[120] Ji W., Zhao M., Wang M., Yan W., Liu Y., Ren S., Lu J., Wang B., Chen L. (2017). Effects of canagliflozin on weight loss in high-fat diet-induced obese mice. PLoS ONE.

[121] Osataphan S., Macchi C., Singhal G., Chimene-Weiss J., Sales V., Kozuka C., Dreyfuss J.M., Pan H., Tangcharoenpaisan Y., Morningstar J. (2019). SGLT2 inhibition reprograms systemic metabolism via FGF21-dependent and -independent mechanisms. JCI Insight.

[122] Day E.A., Ford R.J., Lu J.H., Lu R., Lundenberg L., Desjardins E.M., Green A.E., Lally J.S.V., Schertzer J.D., Steinberg G.R. (2020). The SGLT2 inhibitor canagliflozin suppresses lipid synthesis and interleukin-1 beta in ApoE deficient mice. Biochem. J..

[123] Salim H.M., Fukuda D., Yagi S., Soeki T., Shimabukuro M., Sata M. (2016). Glycemic Control with Ipragliflozin, a Novel Selective SGLT2 Inhibitor, Ameliorated Endothelial Dysfunction in Streptozotocin-Induced Diabetic Mouse. Front. Cardiovasc. Med..

[124] Wang X.X., Levi J., Luo Y., Myakala K., Herman-Edelstein M., Qiu L., Wang D., Peng Y., Grenz A., Lucia S. (2017). SGLT2 Protein Expression Is Increased in Human Diabetic Nephropathy: SGLT2 Protein Inhibition Decreases Renal Lipid Accumulation, Inflammation, and the Development of Nephropathy in Diabetic Mice. J. Biol. Chem..

[125] Huang C.C., Chou C.A., Chen W.Y., Yang J.L., Lee W.C., Chen J.B., Lee C.T., Li L.C. (2021). Empagliflozin Ameliorates Free Fatty Acid Induced-Lipotoxicity in Renal Proximal Tubular Cells via the PPARγ/CD36 Pathway in Obese Mice. Int. J. Mol. Sci..

[126] Thongnak L., Chatsudthipong V., Kongkaew A., Lungkaphin A. (2020). Effects of dapagliflozin and statins attenuate renal injury and liver steatosis in high-fat/high-fructose diet-induced insulin resistant rats. Toxicol. Appl. Pharmacol..

[127] Cai T., Ke Q., Fang Y., Wen P., Chen H., Yuan Q., Luo J., Zhang Y., Sun Q., Lv Y. (2020). Sodium-glucose cotransporter 2 inhibition suppresses HIF-1α-mediated metabolic switch from lipid oxidation to glycolysis in kidney tubule cells of diabetic mice. Cell Death Dis..

[128] Lee Y.H., Kim S.H., Kang J.M., Heo J.H., Kim D.J., Park S.H., Sung M., Kim J., Oh J., Yang D.H. (2019). Empagliflozin attenuates diabetic tubulopathy by improving mitochondrial fragmentation and autophagy. Am. J. Physiol. Renal Physiol..

[129] Hawley S.A., Ford R.J., Smith B.K., Gowans G.J., Mancini S.J., Pitt R.D., Day E.A., Salt I.P., Steinberg G.R., Hardie D.G. (2016). The Na^+^/Glucose Cotransporter Inhibitor Canagliflozin Activates AMPK by Inhibiting Mitochondrial Function and Increasing Cellular AMP Levels. Diabetes.

[130] Feder D., de Fatima Veiga Gouveia M.R., Govato T.C.P., Nassis C.D.Z. (2020). SGLT2 Inhibitors and the Mechanisms Involved in Weight Loss. Curr. Pharmacol. Rep..

[131] Yaribeygi H., Maleki M., Butler A.E., Jamialahmadi T., Sahebkar A. (2023). Sodium-glucose cotransporter 2 inhibitors and mitochondrial functions: State of the art. Excli J..

[132] Rinaldi A., Lazareth H., Poindessous V., Nemazanyy I., Sampaio J.L., Malpetti D., Bignon Y., Naesens M., Rabant M., Anglicheau D. (2022). Impaired fatty acid metabolism perpetuates lipotoxicity along the transition to chronic kidney injury. JCI Insight.

[133] Chen X., Shang L., Deng S., Li P., Chen K., Gao T., Zhang X., Chen Z., Zeng J. (2020). Peroxisomal oxidation of erucic acid suppresses mitochondrial fatty acid oxidation by stimulating malonyl-CoA formation in the rat liver. J. Biol. Chem..

[134] Ndumele C.E., Neeland I.J., Tuttle K.R., Chow S.L., Mathew R.O., Khan S.S., Coresh J., Baker-Smith C.M., Carnethon M.R., Després J.P. (2023). A Synopsis of the Evidence for the Science and Clinical Management of Cardiovascular-Kidney-Metabolic (CKM) Syndrome: A Scientific Statement From the American Heart Association. Circulation.

[135] Martinez-Sanchez F.D., Medina-Urrutia A.X., Jorge-Galarza E., Martínez-Alvarado M.D.R., Reyes-Barrera J., Osorio-Alonso H., Arellano-Buendía A.S., Del Carmen González-Salazar M., Posadas-Sánchez R., Vargas-Alarcón G. (2022). Effect of metabolic control on recurrent major adverse cardiovascular events and cardiovascular mortality in patients with premature coronary artery disease: Results of the Genetics of Atherosclerotic Disease study. Nutr. Metab. Cardiovasc. Dis..

[136] Doroszko A., Janus A., Szahidewicz-Krupska E., Mazur G., Derkacz A. (2016). Resistant Hypertension. Adv. Clin. Exp. Med..

[137] Osorio H., Bautista R., Rios A., Franco M., Santamaría J., Escalante B. (2009). Effect of treatment with losartan on salt sensitivity and SGLT2 expression in hypertensive diabetic rats. Diabetes Res. Clin. Pract..

[138] Bautista R., Manning R., Martinez F., Avila-Casado Mdel C., Soto V., Medina A., Escalante B. (2004). Angiotensin II-dependent increased expression of Na+-glucose cotransporter in hypertension. Am. J. Physiol. Renal Physiol..

[139] Veelen A., Andriessen C., Op den Kamp Y., Erazo-Tapia E., de Ligt M., Mevenkamp J., Jörgensen J.A., Moonen-Kornips E., Schaart G., Esterline R. (2023). Effects of the sodium-glucose cotransporter 2 inhibitor dapagliflozin on substrate metabolism in prediabetic insulin resistant individuals: A randomized, double-blind crossover trial. Metabolism.

[140] Jhuo S.J., Lin Y.H., Liu I.H., Lin T.H., Wu B.N., Lee K.T., Lai W.T. (2023). Sodium Glucose Cotransporter 2 (SGLT2) Inhibitor Ameliorate Metabolic Disorder and Obesity Induced Cardiomyocyte Injury and Mitochondrial Remodeling. Int. J. Mol. Sci..

[141] Kim H.S., Yoon T., Jung C.H., Park J.Y., Lee W.J. (2022). Clinical Efficacy of Sodium-Glucose Cotransporter 2 Inhibitor and Glucagon-Like Peptide-1 Receptor Agonist Combination Therapy in Type 2 Diabetes Mellitus: Real-World Study. Diabetes Metab. J..

[142] Silva I.d.S., Souza L.P., Pereira P.G., Carvalho J.J.d., Moreno A.M., Castro-Faria-Neto H.C., Siqueira R.d.A., d’Avila J.d.C., Carlos A.S. (2022). Combination of a Glucagon-Like Peptide 1 Analog and a Sodium-Glucose Cotransporter 2 Inhibitor Improves Lipid Metabolism Compared to the Monotherapies in Experimental Metabolic Syndrome. J. Endocrinol. Metab..

[143] Shaheer A., Kumar A., Menon P., Jallo M., Basha S. (2021). Effect of Add-On Therapy of Sodium-Glucose Cotransporter 2 Inhibitors and Dipeptidyl Peptidase 4 Inhibitors on Adipokines in Type 2 Diabetes Mellitus. J. Clin. Med. Res..

